# Endoscopic resection of esophageal adenoid cystic carcinoma with submucosal origin and long-term follow-up: a case report

**DOI:** 10.3389/fmed.2026.1722893

**Published:** 2026-03-20

**Authors:** Cuimei Ma, Nana Luo, Yuan Xue, Lingling Niu

**Affiliations:** 1 Department of Gastroenterology, Affiliated Hospital of Jining Medical University, Jining, China; 2 Department of Nephrology, Affiliated Hospital of Jining Medical University, Jining, China

**Keywords:** esophageal neoplasms, adenoid cystic carcinoma, endoscopic ultrasound, endoscopic submucosal excavation, case report

## Abstract

Esophageal adenoid cystic carcinoma (EACC) is an exceptionally rare malignancy where diagnosis by conventional endoscopy remains challenging due to its submucosal growth pattern. We present a case of a 47-years-old male with an esophageal submucosal lesion detected during routine examination. Endoscopic ultrasound (EUS) precisely localized the lesion to the submucosal layer, revealing a hypoechoic mass with a characteristic sieve-like appearance and intact muscularis propria. The tumor was completely resected using endoscopic submucosal excavation (ESE), with histopathology confirming EACC. During 5 years of regular follow-up, the patient maintained excellent condition with no evidence of recurrence or metastasis. This case highlights the critical role of EUS in diagnosing and staging early EACC and demonstrates that endoscopic resection can achieve long-term cure in carefully selected patients, offering a minimally invasive alternative to traditional esophagectomy.

## 1 Introduction

Esophageal adenoid cystic carcinoma (EACC) is an extremely rare malignant tumor, accounting for approximately 0.1% of all esophageal malignancies, with a predilection for males (1, 2). Arising from the submucosal glands of the esophagus, EACC typically grows beneath an intact mucosal layer. This submucosal growth pattern often renders standard endoscopic biopsies non-diagnostic, leading to a high rate of misdiagnosis (3).

Endoscopic ultrasonography (EUS) is indispensable for the early identification and localization of EACC. It accurately determines the layer of origin, displays internal echo features (such as the characteristic sieve-like appearance), and assesses the depth of invasion, thereby providing critical information for formulating treatment strategies (4, 5). While radical esophagectomy has been the traditional standard treatment, endoscopic resection techniques have recently emerged as promising minimally invasive alternatives for early-stage EACC confined to the submucosa without evidence of lymph node metastasis (6).

This report describes a case of EACC that was precisely evaluated by EUS, treated successfully with endoscopic submucosal excavation (ESE), and resulted in long-term recurrence-free survival. It aims to discuss the value of endoscopic minimally invasive therapy for this rare disease.

## 2 Case presentation

A 47-years-old male was admitted to our hospital due to an “esophageal submucosal lesion” discovered during a physical examination. His medical history included hypertension. Physical examination upon admission revealed no significant abnormalities. Laboratory tests, including carcinoembryonic antigen, alpha-fetoprotein, and CA19-9, were within normal limits. Contrast-enhanced computed tomography (CT) of the chest and abdomen showed no abnormalities.

### 2.1 Endoscopic and EUS examination

**FIGURE 1:**
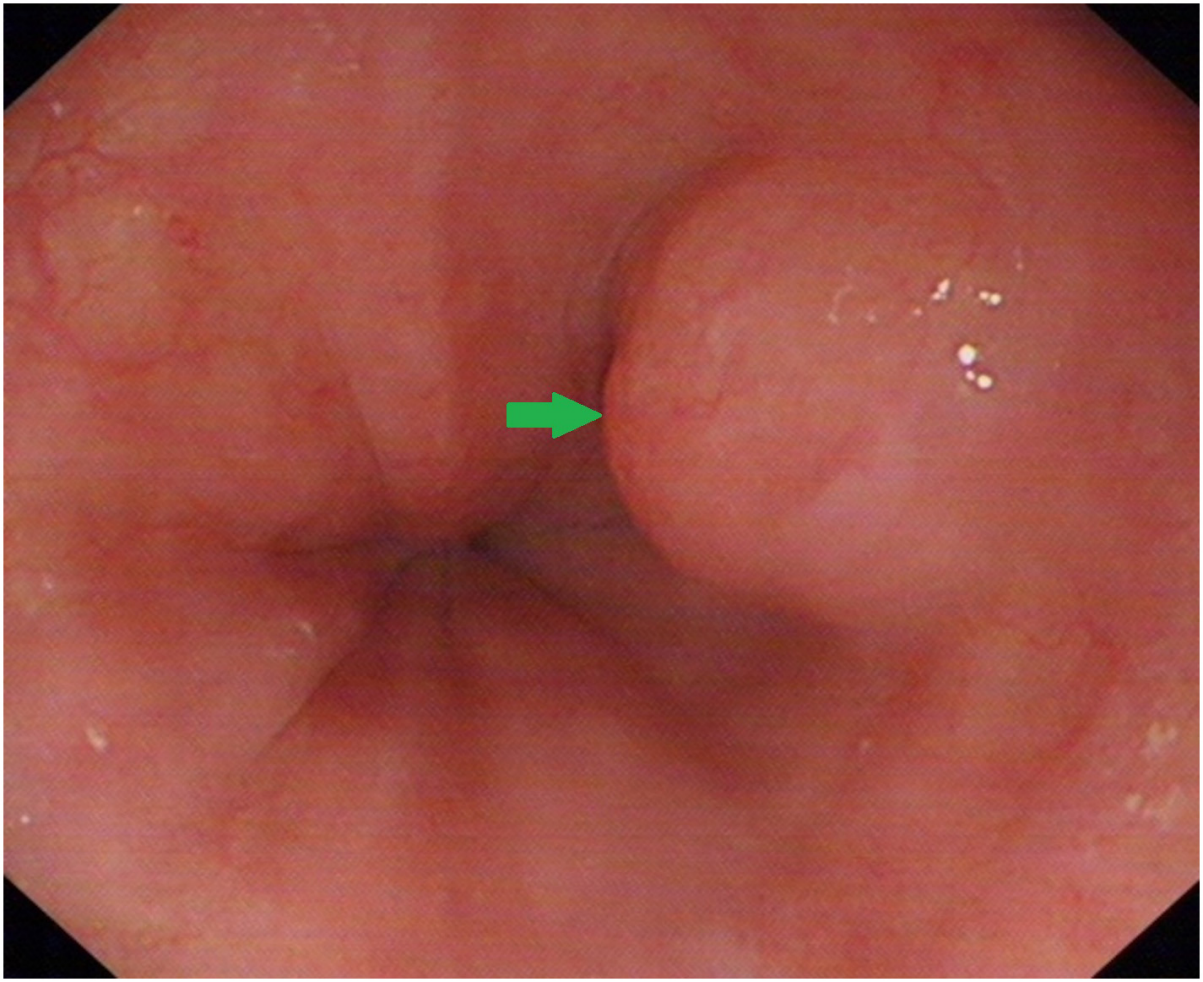
White light endoscopy showing a submucosal lesion in the esophagus with a smooth surface.

White light endoscopy revealed a submucosal lesion, approximately 1.5 cm × 0.8 cm in size, located in the middle-lower esophagus. It had a typical submucosal tumor appearance with smooth overlying mucosa and mildly dilated capillaries at the apex (Figure 1).

**FIGURE 2:**
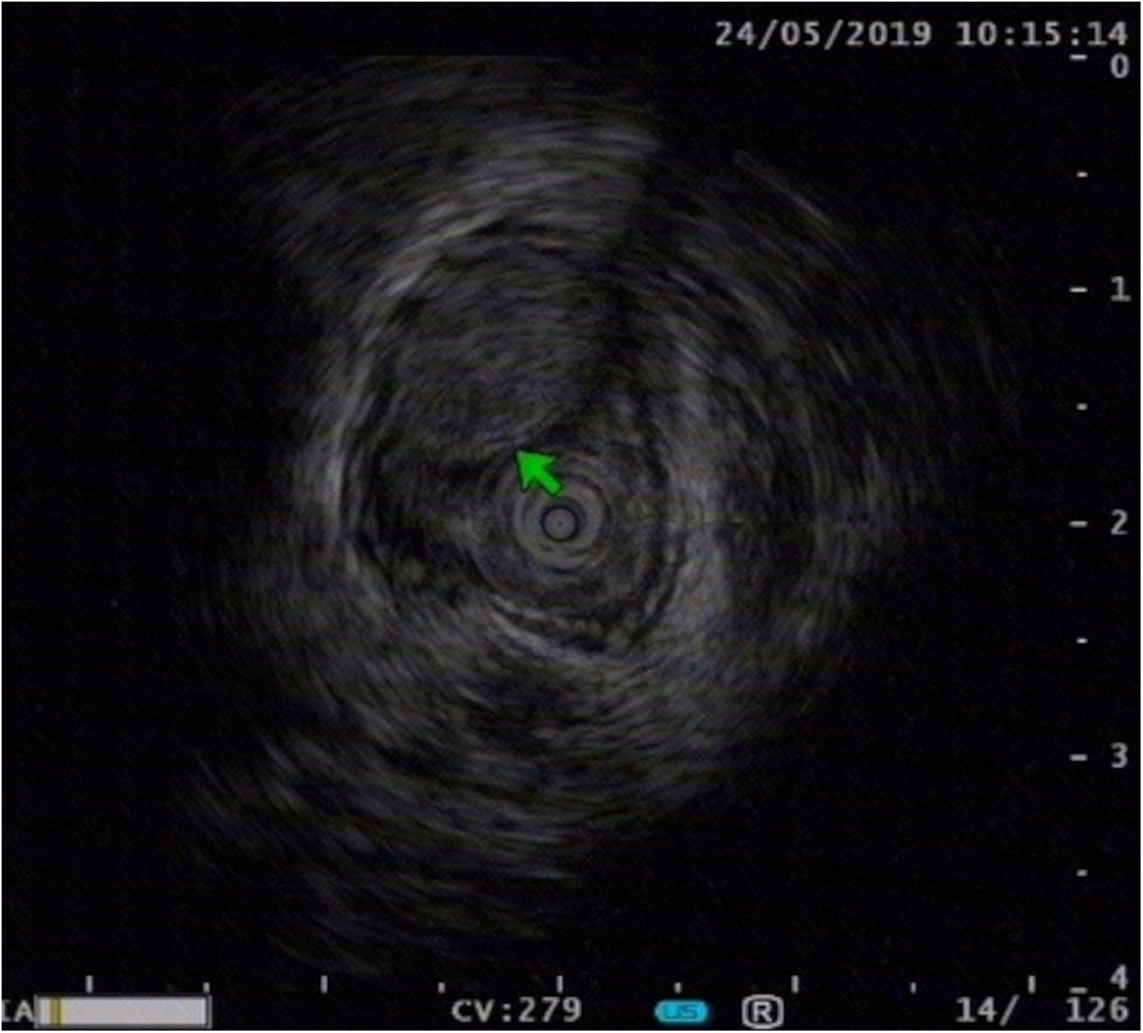
Endoscopic ultrasound revealing a hypoechoic lesion in the submucosal layer with a characteristic sieve-like appearance (arrow). The muscularis propria is intact.

Endoscopic ultrasound examination was pivotal for diagnosis and therapeutic decision-making. EUS clearly showed the lesion originating from the submucosal layer. It appeared as a homogeneous, hypoechoic mass with a characteristic internal “sieve-like” echoic structure. The lesion’s borders were clear, and the muscularis propria was intact and not involved (Figure 2). These EUS findings highly suggested EACC and confirmed its confinement to the submucosal layer.

### 2.2 Preoperative staging and lymph node assessment

Preoperative staging was performed using both endoscopic ultrasound (EUS) and contrast-enhanced CT. EUS systematically examined the periesophageal and mediastinal lymph nodes, with no suspicious features observed (defined as short axis < 1 cm, hyperechoic or isoechoic pattern, and preserved fatty hilum). Similarly, chest and abdominal CT revealed no lymphadenopathy (short axis < 1 cm) or distant metastases. Based on the imaging findings, the tumor was clinically staged as cT1bN0M0 according to the AJCC 8th edition (7).

### 2.3 Treatment process

**FIGURE 3:**
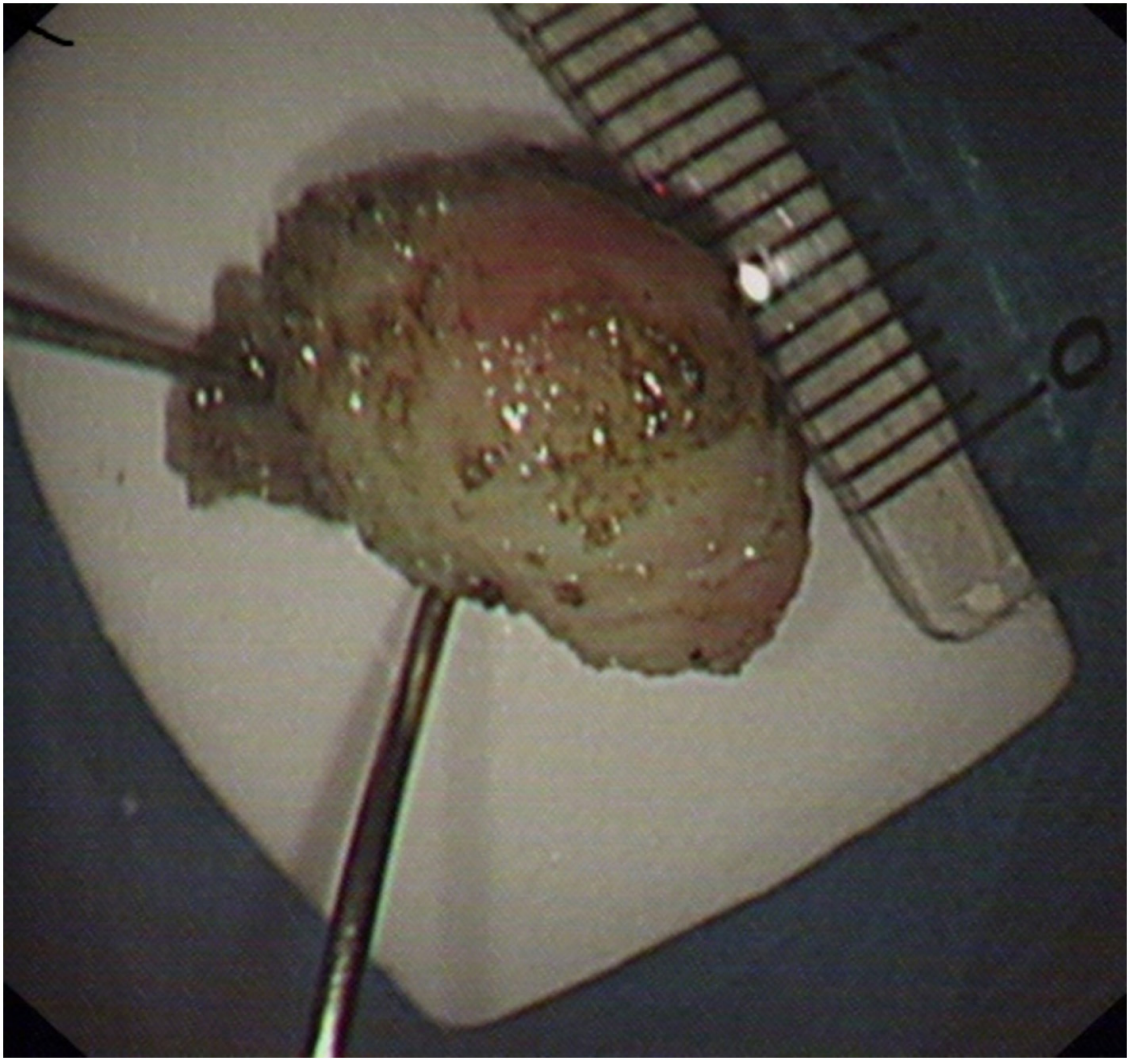
Intraoperative view during endoscopic submucosal excavation showing complete enucleation of the tumor.

After excluding surgical contraindications, the patient successfully underwent ESE under general anesthesia. The procedure involved submucosal lifting, circumferential incision, and meticulous dissection, culminating in the complete enucleation of the encapsulated submucosal tumor (Figure 3) while preserving the muscularis propria. The resected specimen measured 1.2 cm × 0.8 cm × 0.7 cm with a grayish-white, firm cut surface. Hemostasis was achieved, and the procedure was completed without complications.

### 2.4 Pathological findings

**FIGURE 4:**
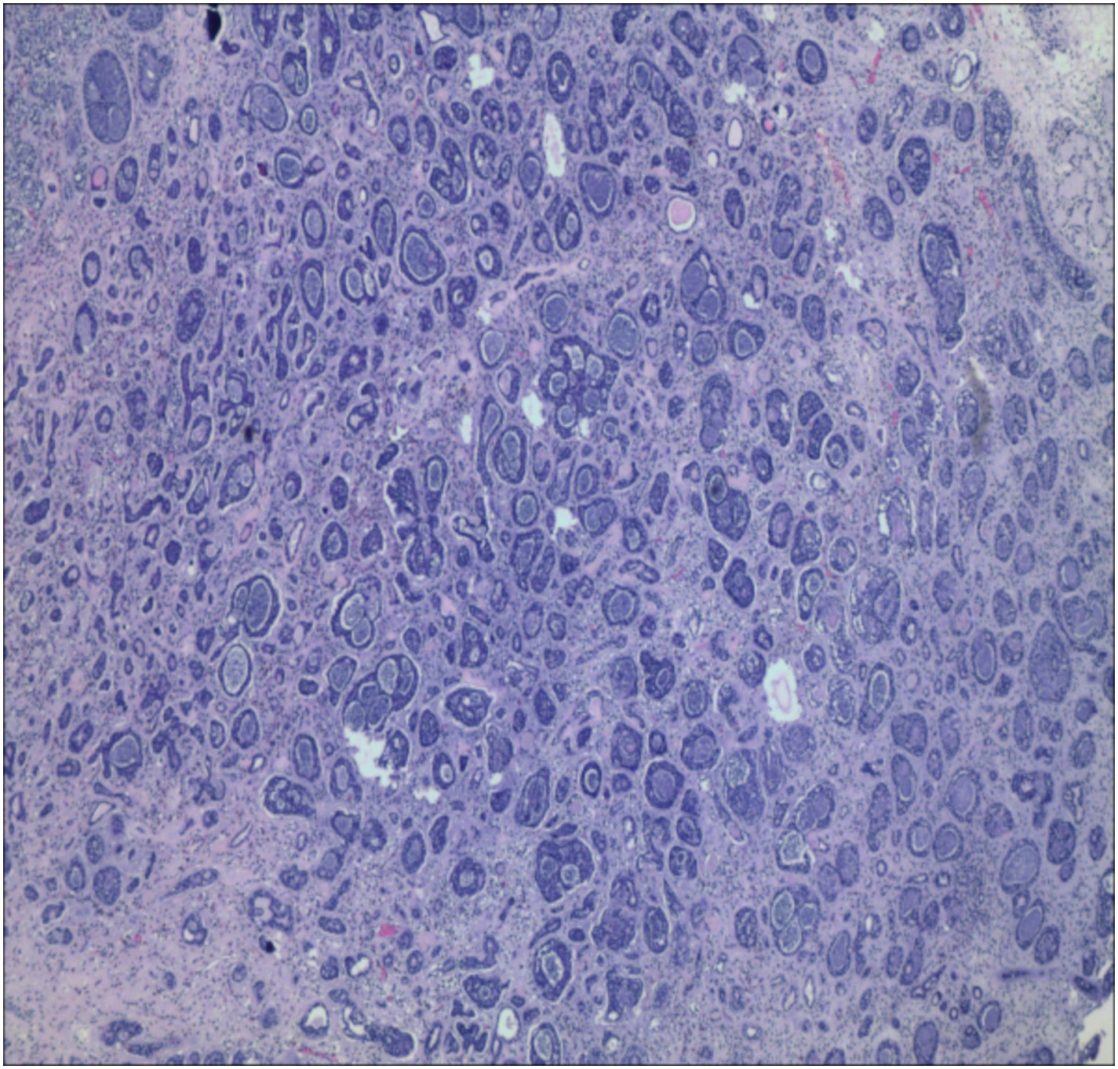
Postoperative histopathology demonstrating the tubular and cribriform patterns characteristic of adenoid cystic carcinoma (H&E staining, ×400).

Postoperative histopathological examination confirmed the diagnosis of esophageal adenoid cystic carcinoma (Figure 4). Immunohistochemical staining results were as follows: CK7 (+), P40 (+), CD117 (+), P63 (+), S-100 (+), SMA (+), and Ki-67 (+, approximately 10%). Histopathological evaluation confirmed complete resection of the tumor with both lateral and deep margins free of carcinoma cells, achieving an R0 resection.

### 2.5 Follow-up

The patient was placed on a stringent and detailed follow-up protocol. Surveillance gastroscopies were performed at 3 months, 6 months, and 12 months postoperatively, and annually thereafter. Surveillance imaging with contrast-enhanced computed tomography (CT) of the chest and abdomen was conducted at 6 months and 12 months after resection, and then annually. As of the last follow-up in August 2025, over 5 years postoperatively, repeated examinations have shown no evidence of local recurrence or distant metastasis. The patient remains asymptomatic and in excellent general condition, confirming long-term disease-free survival.

## 3 Discussion

Esophageal adenoid cystic carcinoma poses significant challenges in diagnosis and management due to its rarity. This case, achieving 5-years recurrence-free survival following EUS-guided ESE, provides valuable evidence supporting endoscopic minimally invasive therapy for early EACC. Given the exceptionally low incidence of EACC, which precludes large-scale randomized trials, each successfully managed case with long-term follow-up holds significant reference value.

The decision to proceed directly to ESE without preoperative biopsy was based on the highly suggestive EUS features of EACC and the recognized limitations of biopsy for submucosal lesions. The characteristic submucosal origin with a sieve-like appearance on EUS provided a high index of suspicion. Biopsy of such lesions through intact mucosa is often non-diagnostic and risks causing fibrosis that can complicate subsequent endoscopic resection. Therefore, for small lesions with typical features, endoscopic resection serves as both a diagnostic and therapeutic procedure, which is an established and efficient management strategy (4).

Endoscopic submucosal excavation enabled complete, R0 resection of the EACC with preserved muscularis propria. The 5-years recurrence-free survival in our case demonstrates its curative potential for early EACC, a finding supported by other reports of successful endoscopic resections (6, 8).

The principal differential diagnoses for an esophageal submucosal lesion are leiomyoma, gastrointestinal stromal tumor (GIST), and neuroendocrine tumor (NET). Endoscopic ultrasound (EUS) is key for differentiation. In our case, the EUS features of a submucosal origin, heterogeneous echogenicity, and a characteristic sieve-like pattern strongly suggested EACC (3). This profile distinguishes it from leiomyoma and GIST, which typically arise from the muscularis propria or mucosa and are homogeneously hypoechoic (4). While esophageal NETs also originate from the submucosa, they lack the sieve-like appearance.

Endoscopic ultrasound holds an irreplaceable position in diagnosing and staging EACC. In our patient, EUS clearly delineated the submucosal origin, homogeneous hypoechogenicity, characteristic sieve-like structure, and intact muscularis propria, findings highly consistent with typical EUS features of EACC reported in the literature (6). The accurate assessment of the lesion’s origin and depth of invasion by EUS directly influences therapeutic decisions: lesions originating from and confined to the submucosal layer are more suitable for endoscopic resection. Furthermore, EACC’s unique biological behavior–characterized by relatively slow growth and a lower rate of lymph node metastasis compared to other esophageal cancers–provides a theoretical basis for local minimally invasive therapy (3).

Traditionally, radical surgical resection has been the primary treatment for EACC. However, for early-stage patients with tumors confined to the submucosa and no evidence of lymph node metastasis, more invasive surgery may not be the only option. This case, along with a few other reported cases (6, 8), confirms that endoscopic resection can achieve complete tumor removal with favorable long-term outcomes. ESE, as an endoscopic technique, is particularly suitable for tumors arising from the submucosal layer (9). The complete resection with negative margins in this case, as confirmed by pathology, indicates that endoscopic resection can achieve curative effects for carefully selected early EACC.

In recent years, endoscopic resection techniques have seen increasingly widespread application in the treatment of gastrointestinal tumors, with continuous refinements in technical maturity (10, 11). The success of this case also benefited from these technological advancements, evidenced by the intact tumor capsule and smooth dissection process, reflecting the operator’s precise mastery of anatomical layers. The absence of recurrence or metastasis during 5 years of regular follow-up further supports the long-term efficacy of endoscopic treatment for early EACC.

The optimal treatment strategy for EACC remains debated. Conventionally, radical esophagectomy is considered the standard therapy but is associated with significant morbidity and impaired quality of life (2). With advances in endoscopic technology and accumulating clinical experience, endoscopic therapy is gradually gaining recognition as a minimally invasive alternative for early, localized EACC without lymph node metastasis (8). The successful experience from this case demonstrates that in carefully selected patients, endoscopic resection can achieve oncological cure while maximizing the preservation of esophageal structure and function, thereby enhancing the patient’s quality of life.

This study has several limitations. It is a single case report, and the follow-up duration, while encouraging, remains relatively limited. Although 5-years recurrence-free survival is persuasive, EACC can exhibit late recurrence patterns, necessitating even longer follow-up to confirm the durability of treatment. Additionally, the lack of preoperative biopsy confirmation and reliance solely on EUS features for decision-making carries inherent risks in clinical practice. However, the high specificity of the EUS findings and the ultimate pathological confirmation support the rationality of the diagnostic and therapeutic pathway chosen in this case. Future studies with larger sample sizes, multi-center collaborations, and extended follow-up data are needed to further establish the role of endoscopic resection in treating EACC.

## 4 Conclusion

This case confirms that patients with early-stage EACC, strictly selected by EUS, can benefit from endoscopic resection, achieving long-term disease-free survival. Endoscopic minimally invasive therapy ensures tumor eradication while better preserving esophageal function and improving quality of life. Future prospective studies are warranted to further define the appropriate patient population, technical standards, and follow-up strategies for endoscopic treatment of EACC.

## Data Availability

The original contributions presented in this study are included in this article/supplementary material, further inquiries can be directed to the corresponding author.
